# Bandwagons or breeding grounds for innovation: What economic elites’ private museums contribute to the arts

**DOI:** 10.1371/journal.pone.0360158

**Published:** 2026-10-09

**Authors:** Johannes Aengenheyster, Olav Velthuis, Alex van Venrooij

**Affiliations:** Department of Sociology, University of Amsterdam, Amsterdam, the Netherlands; Bar Ilan University, ISRAEL

## Abstract

In recent decades, hundreds of private art collectors have opened museum spaces, which they largely finance and control themselves. While such initiatives have been criticized as conspicuous consumption or vanity projects, one public benefit claimed is the display of new artistic perspectives: Due to their strong personal vision and independence, private museums have been argued (including by private museum founders themselves) to showcase underexposed artists and provide artistic innovation. We test this proposition with large-scale exhibition and auction data using Relational Event Modeling. We find that, contrary to claims of artistic novelty, private museums disproportionately exhibit established artists, as their exhibition choices tend to favor artists who have already had longer careers, more prior exhibitions and higher auction turnover. In terms of career status, artists exhibited by private museums correspond most closely to those exhibited by global elite museums. Thus, the potential of private museums to serve as incubators of novel perspectives is limited.

## Introduction

The rise of economic inequality in many countries around the globe [1,2] has resulted in broad political, academic and societal discussions about the extent to which the rich deserve to be rich [3]. These judgments are informed by a range of factors such as political ideology, socio-economic status and cultural influences [4]. Another factor in these judgments is how wealthy people spend their money: when the rich engage in conspicuous consumption, their wealth is more likely to be seen by others as a matter of, e.g., luck and the right connections. But when they engage in philanthropic activities, they tend to be seen as deserving of their wealth because they worked hard for it [5].

Philanthropic giving by the wealthy is however in itself controversial [6], for instance because it would be undemocratic [7], not effective [8], less innovative than it frequently claims [9], and because it would reproduce inequalities [10]. One of the societal domains where this debate over the public benefits of philanthropy and its innovative potential in particular is ongoing, is the arts. Akin to philanthropic initiatives in other realms, wealthy elites frequently donate money and art to public museums [11] – often in exchange for, e.g., their name on display or a place on the museum’s board. Increasingly, members of these elites have opened up their – sometimes massive – private art collections to the public in a museum of their own. Indeed, a new database which we created demonstrates that currently 446 private museums for modern and contemporary art exist around the globe, of which more than 80 percent have been established by wealthy, private art collectors in the new millennium [12].

These philanthropic gestures are controversial, mirroring debates about the merits of elite philanthropy more broadly [13]. On the one hand, some argue that they play crucial, incubating roles for the arts in ways that traditional museums are not willing or able to play, e.g., because of their bureaucratic structures or because public money is involved which comes with strong – and potentially stifling – accountability regimes. One of the public benefits of private museums would be that they foster innovation, discover new talents and serve as vehicles for experimentation [14]. On the other hand, critics argue that their museums would primarily display highly established artists to construct monuments to the collectors’ egos, manifestations of conspicuous consumption and forms of status display [15].

In particular because of a lack of systematic data, claims in either direction have so far gone largely unsupported, with positions often being based on a combination of case studies, impressions and speculation. On the basis of a new large-scale dataset, in this paper we test the innovative capacity of private museums: do they use their financial means and programming independence to showcase emerging and underexposed artists, or do they reproduce the canon of established artists?

In the remainder of the paper we describe the predominant streams of thought that have been developed on this topic in more detail. We then conduct the, to our knowledge, first large-scale empirical investigation of where private museums fall on this emerging-established spectrum using exhibition and auction data. Our results indicate that private museums tend towards the exhibition of artists that have already received a large amount of recognition from other art field institutions: Compared to other museums and exhibition spaces, they are more likely to exhibit artists who are older, have had more prior exhibitions, and have more auction sales and higher auction turnover. Our results thus indicate that, at least in terms of showcasing new artistic talent, the public benefit of private museums is limited.

## Literature review

### Private museums as flexible, idiosyncratic projects

In studies of private museums, different perspectives can be found that speculate about which programming choices private museum founders tend to make as well as the motivations behind these choices. One stream locates the private museum as a responsive, flexible institution headed by a visionary and sometimes idiosyncratic founder. A concise summary of this line of thinking is provided by [14]:

“It has been stressed that private collectors can be more radical, experimental, and faster in their collecting than public institutions where decisions have to be agreed on by boards and committees and are made under the eyes of the public and politicians. They can also look at the otherwise overlooked, explore unique, even idiosyncratic paths. Collectors who aim beyond mainstream or consensus taste can be the speedboats to the tankers or the cruiseships that are public institutions, or pioneers in testing new forms of institutions” (p.14).

These “idiosyncratic paths” can be further differentiated into which art is collected, and how it is presented: In contrast to established museums, which are “primarily devoted to their collection and promoting established artists” [16, p.14] as well as “slow to diversify in the artists they collect” [17, p.69], and “do not focus on collecting contemporary art, which many consider to be a riskier practice” [18, p.881], private museums have been argued to show new artistic talent: “Museums started by private individuals who collect contemporary art often prioritize the showcasing of new or emerging talent” [16, p.14] (see also for Mexico [19] for Russia [20], for China [21]). This would result in “collections [that] are among the most adventurous […] in the world” [14, p.194] (see also [22, p.4]).

Beyond presenting art that previously has received little attention, private museums would also distinguish themselves in the way they present art. Rather than following strict standards, they “have the luxury of being […] unconventional in determining the organisation in which exhibition displays are mounted” [23, p.11], “foster new artistic practices” [17, p.10], “implement innovative display practices” [24, p.84], and “[yield] both innovative building types and new exhibition formats” [14, p.195].

The source of such orientation towards experimentation and novelty is identified in two interrelated structural features of the private museum. First, these museums would follow the unique, novel vision of the founder, leading to a collection with a clear identity and a distinct, personal narrative. Some scholars have in that respect argued that the aim of founders is to provide an alternative to highly institutionalized public museums [25]. In doing so, they seek to develop innovative curatorial approaches that set them apart from public institutions. As Bechtler and Imhof summarize the argument: “What all these institutions share is the visionary spirit of an individual” [14, p.4]. Their “visions” have elsewhere been described as “new” [26, p.16], “unique” [18, p.933], “creative” [18, p.925], “particular” [27, p.52], “specific” (ibid, p.38), “personal” [28, p.157], or “independent”, ([19, p.28] [29, p.62] [26, p.16]).

Second, the fact that these museums are initiated by a single collector or collector couple could mean that they are relatively immune to (changes in) cultural policy, bureaucratic concerns, labor unions, the influence of professional organizations or public sentiments: Whereas “the curators and trustees of public museums make choices about the development of their collections via extensive committee deliberation, private museums can develop swiftly according to the changing desires and interests of the individuals who own them” [15, p.12], also see [17,30,31]. They would also be less tied to conventions when it comes to exhibition making or the need to incorporate public funding goals [19, p.18] (see also [32]). In that respect, the contrast to public museums has been noticed specifically in China, where private museums are seen as relatively autonomous and independent from the government, providing a welcome, flexible alternative to the bureaucratized state system [33, p.45] [34, p.174]. Characterizations of private museum exhibitions and acquisitions processes as independent and flexible (also see [35, p.2] [28, p.171] [36, p.105]) are embraced by private museum founders themselves as well [37, p.1127].

### Private museums as followers of the mainstream and market

This characterization of private museums as unconventional, speedy, visionary and experimental is however heavily disputed. Two distinct counter-arguments have been presented. The first, perhaps paradoxically, also focuses on the personal influence of the collector: Rather than considering them as idiosyncratic visionaries, founders are seen as consumers who lack sophisticated taste of their own [38, p.39], art-historical knowledge [38], a good eye for art [18, p.924], and experience of curation or museum management [39, p.8]. They would be interested mostly in conspicuous consumption, seeking out star architects for their museum design [38]. In that respect, these founders have been categorized as ego-driven and looking for status [14, p.11].

Moreover, the content of the museums would be heavily shaped by existing judgments of what constitutes high-quality art, and would be especially informed by art dealers [15, p.6], auction houses [15] and other market agents [17, p.85], who push the same small group of blue chip artists. As a result, the collections of private museums would be repetitive [40, p.188]. Based on surveys of these institutions, Larry’s List [41], an art market knowledge company, found indeed that “blue-chip artists are by far the most popular collectibles found in private art museums. Andy Warhol is the most widely collected artist, present in 15% of museum founders’ collections” (p.26). The steep rise in the number of private museums has been more generally connected to boom periods in the art market. As Adams observes for China: “the booming art market for Chinese contemporary art, notably around 2010-11, was an additional trigger for collecting and for opening a museum. With prices skyrocketing, owners saw art as a solid investment” [17, p.60]. In their preference for ’blue chip artists’, private museums would be different from public museums, which would frown upon those artists and see them as too commercial and instead seek out artists whose artistic merit is demonstrated by institutional rather than market recognition [15]. In that sense the different positioning of private and public museums can be captured by the Bourdieusian notion of the heteronomous and autonomous poles of the field of cultural production [42].

Alternatively, private museums would tend to follow the exhibition choices of established institutions with international reach. As a result, their collections would usually be mainstream, veering towards the popular, and avoiding difficult curatorial choices [19, p.61]. The most systematic quantitative study of exhibition choices so far, comparing French private foundations to public museums, finds that public museums tend to promote French artists (as they are required to do so by public policy), while private foundations in France focus on international artists [43]. In doing so, “they participate in reinforcing the common belief in the existence of a hierarchy of legitimate artists, at the top of which are American artists (followed by German and English ones)” (p.127). Furthermore, whereas public museums have “diversified portfolios” (p.124), showing artists at a wide range of career stages and countries of origin, private foundations have “stratified portfolios” dominated by “international stars” and “rising foreign artists” (p.123). The focus of private museums on exhibiting international (star) artists, to the detriment of exhibition chances of local artists, has also been observed in Mexico [35] and China [44].

The second argument against the idea of private museums as innovative and experimental likewise claims that private museums focus on established artists. However, in this case, the reasoning does not hinge on the founder’s poor taste, reliance on market actors, or status-driven motivations, but on financial considerations: according to these scholars, private museums focus on established artists and organize blockbuster exhibitions around them in order to cover museum operation costs. This, in turn, is necessary given that they usually have no stable funding sources from governments or donor pools, and given that the founder has to cover expenses not covered by operations (which can be substantial as on average earned income covers less than 50% of expenses [45, p.26]). As one scholar argues: “’Blockbuster’ exhibitions guarantee that the museum will earn money, because the preferences of a larger group of people are taken into account” [46, p.16].

These two opposing arguments which scholars of private museums have developed, which position private museums either as promoters of emerging art or as reproducers of already highly consecrated artists, can be related to broader theoretical perspectives. The first ties in with long-standing traditions of theorizing elites as versatile, discerning, innovative, entrepreneurial, and in particular in domains like philanthropy, transferring such skills to other areas where they are active, which results in innovation [47]. The second with equally long-standing traditions of theorizing the elites as engaged in status display via conspicuous forms of consumption [48] or with a critique of taste as presented by the French sociologist Pierre Bourdieu [49], who saw the field of culture as a field where different factions of the elite struggle for domination, and argued that in this struggle new wealthy elites lack the appropriate cultural capital.

In the following analysis, we will test both arguments by scrutinizing the exhibition choices of private art museums: are the artists that they exhibit novel, in the sense that they have not had much exposure to the public before, and lack an active commercial market? Or are they, by contrast, focusing on established and commercial artists?

## Data

We combine several data sources to investigate the exhibition choices of private museums, and how these choices differ from those of other institutions. In particular, we use:

- A database of private museums, listing current and prior private museums.
- The Artfacts database on exhibition choices (artists) of a large number of art institutions.
- The Art Newspaper Museum ranking, a yearly ranking of the world’s most visited museums.
- The Lotsearch auction database, which describes artist-year auction turnover.

These databases provide three crucial components, namely exhibition choices (Artfacts), institution type (Private museum database, Artfacts, Art Newspaper Museum Ranking), and artist career statistics (Artfacts, Lotsearch). We describe each database and relevant processing steps.

### Database of private museums

This study is based on a new database of private art museums [12]. A private museum is defined as “an art institution owned or governed by one or several private persons, which receives no or limited public funding, has a permanent collection of modern and/or contemporary art, and makes this collection accessible to the public, in a building or physical structure, on an ongoing basis” (p.7). To document the development of private museums, existing databases of private contemporary art museums [41,50,51] were combined; additionally web research was conducted by searching systematically for the term “private museum”, “private art museum” and “private contemporary art museum” in a number of online art media (The media sources used were: Artforum, Artnet, Art Territory, Artsy, My Art Guides, Artnews, Artfcity, Frieze, The Art Wolf, The Art Newspaper, Art Privee, Widewalls, White Hot Magazine and Hyperallergic). After recording the names and deleting duplicates, it was investigated using publicly available information whether the organization corresponds to the employed definition of a private museum. Currently, the database includes 550 museums (of which 453 are currently open, the remainder having closed or transformed to the extent that they no longer constitute a private museum), which are located in 65 countries, with the majority being located in Western Europe (136), East Asia (97) and North America (77). The majority of them (429, or 78%) have been opened from 2000 onwards.

### Artfacts: Exhibition choices

We furthermore use the database of Artfacts, an art-market knowledge company specialized in exhibition information. The entire database includes around 1.2 million exhibitions and 1.1 million artists [52]. We use a subset that focuses on artists born after 1945 whose demographic information is complete and who have had at least 10 exhibitions; below this number, an artist may not be considered professional. The dataset includes

- 39 471 artists; including information on gender, year of birth and nationality (or nationalities).
- 544 982 exhibitions; including information on the organizing institution, start and end date.
- 1 740 124 artist-exhibition links; as often multiple artists are exhibited in a group exhibition, this exceeds the number of exhibitions.
- 37 151 institutions; including location and institution type.

As our goal lies in comparing the exhibition pattern of private museums to those of other institutions, we combine Artfacts own classification system of 23 different institution types into a typology of four categories, namely private galleries (commercial organizations representing and selling the works of their roster of artists), events (such as art fairs and biennials), public institutions (which is a single category in the Artfacts classification system) and other exhibition spaces (including what Artfacts labels “non-profit organizations”, “associations” and “exhibition spaces”). The latter two, public institutions and other exhibition spaces, are of primary interest, as like private museums, their primary activity is the non-commercial display of art. The differentiating criterion between public institutions and other exhibition spaces is that public institutions possess a substantial permanent collection [53,54]; they are therefore also referred to as “museums” in other studies [55] (the word “public” refers primarily to public orientation, accessibility and impact and does not imply governmental governance or funding, and hence this category contains different legal forms such as state-run, non-profit or corporate organizations). Public institutions include regional and national flagship institutions, whereas other exhibition spaces predominantly refer to smaller local art spaces, centers or venues without permanent collections.

We then looked up all private museums from our database in Artfacts and labeled them as a separate fifth category of institutions. 337 (61.3%) of the private museums were found to have exhibitions in the Artfacts database; this group in total organized 5645 exhibitions (around 1% of all exhibitions in the Artfacts dataset).

### The Art Newspaper museum ranking: Top-Tier museums

While the “public institutions” as defined by Artfacts have been used as equivalent to museums, they nevertheless constitute a large and heterogeneous group. To also obtain a contrast to the most well-known museums (which likely often constitute the implicit comparison group to private museums in the arguments about private museum exhibition choices described in the literature review), we use the museum ranking published annually by The Art Newspaper to create a sixth category of top-tier museums. Position in the ranking is defined by visitor numbers: Since the early 1990s, The Art Newspaper has sent annual surveys about visitor attendance to a wide range of major institutions, and based on them publishes a report listing the 100 most visited institutions (until 2007, visitor numbers were only reported by exhibition, and have hence been aggregated to the institution level). We retrieved all rankings published between 2000 and 2024 (except for the ones published in 2006 and 2017, which we could not find; however the top 70 entries of 2017 were retrieved from the Wikipedia archive). These gaps of 2006 and 2017 were filled using records from the previous year, thus 2006 positions were based on 2005 values, and the bottom 30 of 2017 were based on 2016 data. In total, 403 institutions appear in the Art Newspaper rankings at least once, of which 320 have exhibition activity in Artfacts (the remaining 83 institutions are not in the Artfacts database because they focus on, e.g., natural or national history, technology, or art other than contemporary art). To ensure focus on elite institutions, we define a museum as top-tier only if it appears at least ten times in the ranking, resulting in 81 institutions. This category thus includes the most famous institutions such as the Museum of Modern Art, the Whitney Museum of American Art (New York), the Tate Modern (London) or the Centre Pompidou (Paris). One institution, the Saatchi Gallery (London), fulfills the criteria of both a private and a top-tier museum; due to our interest in private museum choices vis-a-vis other organizational forms it is classified as a private museum.

### Merging and filtering steps

With our six institutional forms defined (galleries, events, other exhibition spaces, public institutions, top-tier museums and private museums), we merge artist and institution information to the artist-exhibition links (N = 1 740 124) and perform a number of filtering steps: First, a small number of institutions belonging to organizational forms that do not fit into our institution classification system, such as auction houses, artist studios, schools or journals are excluded. Secondly, artists who had their first exhibitions before the age of 16 are excluded, as these often appeared to be data errors. The final dataset consists of 1 588 392 artist exhibition links (96.9% of the original) based on 511 360 exhibitions (98.1% of original) and 38 912 artists (98.6% of original) by 33 189 institutions (96.7% of original).

Although less than 5% of the artists of the complete Artfacts database are included, these account for almost 50% of the exhibitions. This winner-takes-all nature of the art field [56] also persists in the artist subset, with the top 10% of artists accounting for 39% of the exhibitions.

Since the exhibition data is collected as part of the commercial activities of Artfacts, rather than for scientific purposes, data quality cannot be taken for granted. Indeed, manual comparison of Artfacts exhibition records with exhibition records published on artists’ personal websites indicated occasional coverage gaps. Systematic data quality issues can also arise from variation in data collection efforts over time, leading to better coverage for some periods rather than others. We will address these issues via Relational Event Modeling.

### Lotsearch auction data

To document the performance of artists on the secondary auction market, we use auction data by Lotsearch [57], a website tracking the auction market. A publicly available API was used to collect aggregate artist-year information, in particular the number of sales and average sale price per artist year, which allows us to construct a measure of yearly auction turnover as their product. In total, information of 2 856 314 auction sales by 61 609 artists was collected. We consider this indicator not as a complete account of artist auction sales, but as an indicator of market success, i.e., as proportional to the recognition and demand that artists receive from auction participants such as art collectors and institutions. 4614 artists (11.9%) included in our sample of the Artfacts database are covered by Lotsearch, again indicating the winner-takes-all market in which only a small section of artists is popular enough to have an active auction market. These artists have accumulated 185 380 auction sales with a total turnover of 13.7 billion euros.

## Methods

We first describe the institutions, artists and artist-exhibition links by presenting summary statistics, both overall and by institution type. We focus in particular on artist career characteristics to see whether private museums tend to exhibit emerging or established artists. While such statistics do not cover the entire range of arguments made about private museum exhibition choices, in particular those that refer to mode of presentation (i.e., the argument that private museums exhibit artists in new ways), and do not cover the full breadth of artistic display such as aesthetic content or styles, they provide substantial insights that address many proposed relationships, in particular whether private museums choose to display artists already consecrated by other institutions or tend towards underexposed positions. We then use Relational Event Modeling [58] to more robustly model the exhibition choices of private museums and other institution types.

## Dataset composition

Table 1 shows the distribution of the categorical, time-invariant variables (artist gender and nationality, institution type and location) of the entities (artists, institutions) and the artist-exhibition links. It can be seen that the dataset consists disproportionately of artists who are male (62.7% of artists, 64.7% of artist-exhibition links) and European (61.9% of artists and 62% of artist-exhibition links), reflecting the male-dominated and Eurocentric character of the contemporary art world [59,60]. European institutions are also strongly represented in the dataset (with 66.4% of institutions located in Europe which account for 67.6% of artist-exhibition links). In terms of institution type, galleries, public institutions and other exhibition spaces account together for more than 90% of institutions and artist-exhibition links. Private museums make up only 0.93% of the institutions, but show disproportionately many artists as they account for 2.2% of artist-exhibition links. Top-Tier museums are even more active: While constituting only 0.23% of institutions, they organize 1.6% of artist-exhibition links.

**Table 1:** Categorical variables by entity type and Artist-Exhibition link.

	Entity		Artist-Exhb. Link	
	Count	Percent	Count	Percent
**Artist – Gender**				
Female	14.1 K	35.7%	541.8 K	34.1%
Male	24.7 K	62.7%	1.0 M	64.7%
Other	631	1.6%	18.9 K	1.2%
**Artist – Nationality**				
Africa	918.0	2.3%	38.8 K	2.4%
Asia	4.5 K	11.4%	166.5 K	10.5%
Europe	24.4 K	61.9%	984.2 K	62.0%
Latin America	2.5 K	6.2%	93.4 K	5.9%
North America	6.4 K	16.3%	289.4 K	18.2%
Oceania	1.0 K	2.6%	35.4 K	2.2%
**Institution – Type**				
Event	1.8 K	5.0%	86.5 K	5.4%
Gallery	17.5 K	48.6%	633.2 K	39.9%
Other Exhibition Space	8.9 K	24.7%	258.6 K	16.3%
Public Institution	7.4 K	20.5%	549.6 K	34.6%
Private Museum	336.0	0.9%	35.7 K	2.2%
Top-Tier Museum	81	0.2%	24.9 K	1.6%
**Institution – Location**				
Africa	394	1.1%	12.9 K	0.8%
Asia	3.4 K	9.4%	108.4 K	6.8%
Europe	23.9 K	66.4%	1.1 M	67.6%
Latin America	1.5 K	4.2%	47.4 K	3.0%
North America	6.1 K	17.1%	307.4 K	19.4%
Oceania	675	1.9%	38.1 K	2.4%

Table 2 shows descriptive statistics of exhibition choices and artist careers. Artists on average start their career in their early thirties, and are observed on average for 19.5 years. Over this time span they participate in an average of 1.87 exhibitions per year and accumulate an average of 40.4 exhibitions by the end of the observation period.

**Table 2:** Artist career summary statistics.

Measure	Mean	SD	Median	Min.	Max.
Age (years) at beginning of career	32.4	7.7	31.0	17.0	71.0
Career length (years) at end of observation period	19.5	8.8	18.0	0.0	56.0
Exhibitions per year	1.9	3.1	1.0	0.0	84.0
Exhibition count at end of observation period	40.4	59.5	24.0	4.0	1.7 K
Number of prior exhibitions at exhibition	62.7	114.7	25.0	0.0	1.7 K
Age (years) at exhibition	44.3	10.8	43.0	17.0	76.0
Age (years) at end of observation period	51.9	11.3	51.0	21.0	76.0
Cum. auction turnover (€) of artist at exhibition	1.2 M	26.0 M	0.0	0.0	2.3 B
Cum. auction turnover (€) at end of observation period	291.9 K	13.8 M	0.0	0.0	2.3 B

Exhibitions are distributed extremely unevenly: At each new exhibition, artists have had on average 62.7 previous exhibitions; the average artist chosen for exhibition has therefore had more prior exhibitions than the average artist has had over the entire observed period (40.4). This difference is due to concentration of exhibitions in the careers of a relatively small share of artists: As (super)stars are exhibited frequently, they have accumulated large numbers of prior exhibitions (the artists with the highest number of exhibitions over the observation period include Cindy Sherman (1719), Damien Hirst (1115), and Kiki Smith (1105)), and their high exhibition rate results in their high prior exhibition counts contributing disproportionately to the average of prior exhibitions at exhibition.

Substantial inequality can also be seen by median values being lower than average values, and standard deviations being higher than averages. Auction turnover is especially concentrated, partly due to only 4614 artists having any turnover: while most artists never see their work sold at auction, the works of a small minority are bought and sold for sums ranging up to tens of millions of euros.

The discrepancy between prior exhibition count at exhibition and exhibition count at the end of the observation period is also indicative of a Matthew effect [61,62] in exhibition choices: Institutions do not choose artists independently of their exhibition count, but especially select those that have already been frequently exhibited before. We now turn to institutional variation in this exhibition pattern.

### Career statistics by organizational form

Fig 1 compares different types of institutions by plotting the distribution of three career characteristics of exhibited artists: career year, count of prior exhibitions and cumulative auction turnover. The top row shows the distributions for each institutional form. The bottom row shows the percentage of exhibitions for each form over the range of the career characteristic. Of particular interest is the comparison between private museums on the one hand, and public institutions, other exhibition spaces and top-tier museums on the other hand.

**Fig 1:**
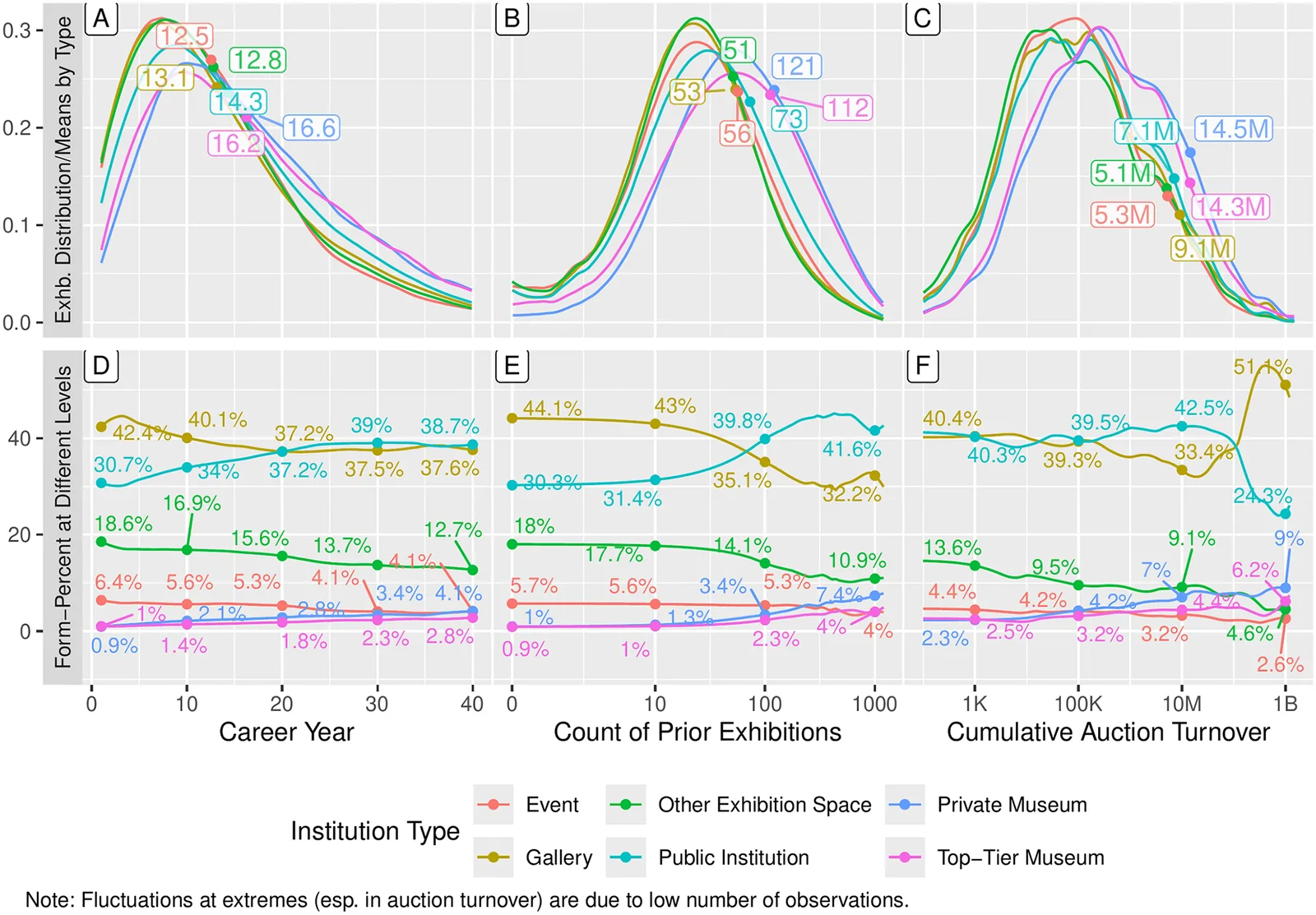
Distribution of exhibited artists by institution type and career variables.

Overall, private museums can be seen to, on average, exhibit artists who are more established than those shown in public institutions and other exhibition spaces, with higher averages and right-shifted distributions (upper row), and hence a (relatively) strong presence at the upper end of the emerging-established spectrum (bottom row). In fact, the distribution of artists shown in private museums resembles most closely that of top-tier institutions, which also disproportionately show established artists.

More specifically, in terms of career year, private museums tend to show artists who, on average, have already had longer careers at the time of exhibition (16.6 years in private museums and 16.2 in top-tier museums vs 14.3 in public institutions and 12.8 in other exhibition spaces; panel A). Moreover, panel D shows that whereas only around 0.96% of the shows of artists in their first three years take place in private museums, private museums organize 3.4% of the shows involving artists in the 30th career year, an increase by 255%. Public institutions (and top-tier museums) likewise have a preference for established artists, increasing from 31% (0.98%) of shows of artists in the first years of their career to 39% (2.3%) of artists in their 30th year, yet these increase (27% and 132%) are less pronounced than for private museums.

Panel B shows again that private museums’ exhibition choices tend to favor artists who have already been substantially exhibited elsewhere. Whereas the average artist in a private museum has had 121 prior exhibitions, the average artist exhibited in a public institution has been exhibited 73 times before. Again, private museums are most similar in their choices to top-tier museums, where chosen artists have on average been shown 112 times before. Panel E shows a relatively strong presence of private museums at exhibitions of artists with higher prior exhibition counts. While private museums organize 2.2% of all exhibitions, they only account for around 1% of early career shows, but around 7.4% of shows involving superstars with 1000 shows.

Information on auction turnover is shown in Table 3 and Fig 1, panel C and F (since only around 11.9% of artists have any auction turnover, the distribution only includes artist-exhibition links of artists who have had prior turnover). Around 21.7% of artists exhibited in private museums have had their works sold at auction the year before (similar to 22.9% in top-tier museums), whereas this number is substantially lower for public institutions, where only 11.7% of exhibited artists were previously sold at auction. A similar ratio is present for the percentage of artists who were ever sold at auction, which is the case for 29.1% for artists exhibited in private museums and 17.6% for artists exhibited in public institutions (30.7% for top-tier museums). Average cumulative turnover volume is also higher for artists shown in private museums than in public institutions, both when including all artists (4.2 million Euro vs 1.2 million Euro, the high values for private museums being partly due to more showing artists with non-zero turnover) and only those whose works were sold at auction before (14.5 vs 7.1 million Euro). While private museums do not exclusively show auction darlings, they do seem to value auction success more than the average public institution.

**Table 3:** Auction information by institutional form.

	% with any turnover		Avg. Cum. turnover	
	prev. year	ever	all artists	prior turnover > 0
Other Exhibition Space	5.8%	9.5%	479.6 K	5.1 M
Public Institution	11.7%	17.6%	1.2 M	7.1 M
Private Museum	21.7%	29.1%	4.2 M	14.5 M
Top-Tier Museum	22.9%	30.7%	4.4 M	14.3 M
Gallery	9.5%	14.5%	1.3 M	9.1 M
Event	6.5%	10.7%	563.7 K	5.3 M

### Gender and nationality

In terms of artist demographics, variation by institutional form can be observed. Men are overrepresented among artists (62.7%) and exhibition choices (64.7%), and in particular among private museums (67.5% of exhibited artists are male, compared to 63.7% in public institutions and 64.3% in top-tier museums). Private museums moreover have a preference for artists from outside the country in which the institution is located. In private museums 63.0% of artists shown are international, a proportion substantially higher than in public institutions (50.4%) and even top-tier museums (56.2%).

### Relational event modeling

While these descriptive statistics give a clear indication that private museums tend to exhibit established artists to a greater degree than public institutions and other exhibition spaces, they are not able to address confounding (i.e., estimation of association while holding other variables constant), compare effect sizes and account for composition aspects (as private museums have emerged only relatively recently, the bulk of their exhibitions might take place in time periods where many artists have established careers and private museums are therefore not disproportionately exhibiting established artists) and data coverage variability issues. To address these limitations, we use Relational Event Modeling (REM [58,63]).

REM can be understood as an application of event history [64] to network dyads, i.e., tie formation between nodes in a network [65, p.100]. It has been used in various domains such as email networks [66], interstate conflict [67] or interlocking board directorates [68]. In our case, exhibitions can be modeled as a bipartite network of two classes of actors (institutions and artists), with events (exhibitions) creating ties between institutions and artists (but not other institutions). Our primary interest is whether artist characteristics (such as their number of prior exhibitions) or dyad characteristics (such as whether artists were previously exhibited at the institution in question) increase or decrease the probability of the appearance of a tie, i.e., a particular artist having an exhibition at a particular institution, as well as whether these effects differ between institution types.

REM assesses these effects by comparing events that took place (the event set) with those that could have taken place (the risk set); in terms of exhibitions this corresponds to comparing the artists that were exhibited to those that could have been chosen instead. REM thus closely corresponds to the curatorial decision-making process in which the choice of exhibiting a certain artist is made in the context of other artists having possibly been chosen as well. Due to our primary interest in the variation of preferences between institutional forms, we use a sender-stratified REM [69] where all alternatives of the observed event involve the institution that organizes the event in question (this approach allows separation of preferences from the rate at which different institution types organize exhibitions). Intuitively, a sender-stratified REM analyzes how, given that a specific institution organizes an exhibition, features of the candidates (and candidate-institution features) are related to the chance of being chosen, i.e., which features differentiate the observed exhibition choice from the candidates which could have been chosen but were not. Technically, in sender-stratified REM the risk set consists of all other available artists (up to 38 912 per event), whereas in unstratified REM [70], the risk set would consist of up to 1.1 billion dyads (38 912 artists * 33 189 Institutions).

Even though sender-stratification reduces the risk set, it is still extremely computationally expensive as for all 1 588 392 events, the entire artist risk set of up to 38 912 artists would have to be evaluated, resulting in a dataset size of tens of billions of ties. We thus use several sampling procedures to reduce the calculations to manageable proportions. First, we exclude exhibitions in galleries and events, and focus only on exhibitions in other exhibition spaces, public institutions, private museums and top-tier museums, as these constitute the primary venues for non-commercial display of art. Second, we also only focus on exhibitions taking place between January 1, 2000 and December 31, 2021. Third, we limit the number of total events: We use all events in top-tier (20 655) and private museums (33 419) as these organize a much smaller share of total events, but constitute a main area of interest, and sample 33 419 (the larger number of events of the two smaller institution types) events from public institutions and other exhibition space types, resulting in a total of 120 912 events. Fourth, we use case-control sampling [71]: Rather than evaluating the entire risk set for each event, we randomly sample for each event a number of non-events (i.e., artists who were not shown). Case-control sampling has been applied to REM [65,70], with reliable parameter estimation already achievable with one control per case. We here use 25 controls per case, i.e., in every exhibition, the artist who was chosen is compared to 25 that were not chosen by that institution, resulting in a total dataset size of 3 143 712.

The dataset thus consists of the following variables:

- The dependent variable, Exhibition: binary indicator whether an exhibition takes place, i.e., whether a tie between the artist and institution in question is observed
- Main independent variables:
  - Count of prior exhibitions (log): Number of prior exhibitions in all institution types (log) by the artist up until the time of exhibition.
  - Auction performance (log): logged cumulative auction turnover up to the year before the exhibition.
  - Career year: Number of years passed since first exhibition
  - Institution Type: Other exhibition space (reference category), public institution, top-tier museum or private museum
- Control variables:
  - Gender: Female (reference category), Male, or Other.
  - International Exhibition: a dyad characteristic which measures whether any of the artist’s nationalities differs from the location of the institution
  - Repeated exhibition: Whether the artist has previously been exhibited at the institution of the event.
  - Event fixed effects, which are used in case-control sampling to compare within the (sampled) risk set of each event [65].

All independent variables (prior exhibitions, auction performance, career year, gender, international exhibition and repeated exhibition) are interacted with institution type (private museum, top-tier museum, public institution or other exhibition space) to estimate the effect for each independent variable for each institution type. Coefficient estimation is conducted via Poisson regression (equivalent to Cox piece-wise exponential hazard model or conditional logit models [72,73]).

### REM results

Table 4 shows the regression results (original scale in Model 1, standardized in Model 2). Fig 2 plots the slopes (marginal effects) of the main independent variables (standardized) by institution type, and Fig 3 shows average adjusted predictions. Coefficients refer to multipliers of the baseline hazard rate, i.e., a coefficient of 1 corresponds to an e = 2.71-fold increase in the probability of an artist being chosen given a 1-unit increase in the independent variable. Thus, if we compare two artists, A and B, with A scoring 1 unit higher on an independent variable with a coefficient of 1, A’s exhibition chances are 2.71 times higher than those of B. The models have substantial explanatory power as McFadden’s pseudo-R^2 reaches 0.31, indicating that the independent variables substantially predict exhibition chances (see the supplementary materials for a more extensive discussion). Overall the REM results support the descriptive findings of private museums disproportionately exhibiting established artists, but also provide additional insights.

**Table 4:** REM models.

Variable	Model 1 (original scale)	Model 2 (standardized)
# prev. Exhibitions (log)	.77 (0.01)^*^|^*^|^*^	.85 (0.01)^*^|^*^|^*^
# prev. Exhibitions (log) * Ins. type (Private Museum)	.49 (0.01)^*^|^*^|^*^	.54 (0.01)^*^|^*^|^*^
# prev. Exhibitions (log) * Ins. type (Public Institution)	.23 (0.01)^*^|^*^|^*^	.25 (0.01)^*^|^*^|^*^
# prev. Exhibitions (log) * Ins. type (Top-Tier Museum)	.49 (0.01)^*^|^*^|^*^	.53 (0.02)^*^|^*^|^*^
Auction turnover (log)	.00 (0.00)	.00 (0.01)
Auction turnover (log) * Ins. type (Private Museum)	.04 (0.00)^*^|^*^|^*^	.11 (0.01)^*^|^*^|^*^
Auction turnover (log) * Ins. type (Public Institution)	.01 (0.00)^*^|^*^|^*^	.04 (0.01)^*^|^*^|^*^
Auction turnover (log) * Ins. type (Top-Tier Museum)	.03 (0.00)^*^|^*^|^*^	.09 (0.01)^*^|^*^|^*^
Career Year	−.07 (0.00)^*^|^*^|^*^	−.59 (0.01)^*^|^*^|^*^
Career Year * Ins. type (Private Museum)	−.00 (0.00)	−.02 (0.01)
Career Year * Ins. type (Public Institution)	.02 (0.00)^*^|^*^|^*^	.17 (0.01)^*^|^*^|^*^
Career Year * Ins. type (Top-Tier Museum)	.02 (0.00)^*^|^*^|^*^	.14 (0.02)^*^|^*^|^*^
Gender – Male	−.13 (0.01)^*^|^*^|^*^	−.13 (0.01)^*^|^*^|^*^
Gender – Male * Ins. type (Private Museum)	.17 (0.02)^*^|^*^|^*^	.17 (0.02)^*^|^*^|^*^
Gender – Male * Ins. type (Public Institution)	.03 (0.02)	.03 (0.02)
Gender – Male * Ins. type (Top-Tier Museum)	.02 (0.02)	.02 (0.02)
Gender – Other	.10 (0.06)	.10 (0.06)
Gender – Other * Ins. type (Private Museum)	.02 (0.09)	.02 (0.09)
Gender – Other * Ins. type (Public Institution)	−.06 (0.08)	−.06 (0.08)
Gender – Other * Ins. type (Top-Tier Museum)	.08 (0.09)	.08 (0.09)
Repeated exhibition	4.02 (0.04)^*^|^*^|^*^	4.02 (0.04)^*^|^*^|^*^
Repeated exhibition * Ins. type (Private Museum)	−.42 (0.05)^*^|^*^|^*^	−.42 (0.05)^*^|^*^|^*^
Repeated exhibition * Ins. type (Public Institution)	−1.15 (0.05)^*^|^*^|^*^	−1.15 (0.05)^*^|^*^|^*^
Repeated exhibition * Ins. type (Top-Tier Museum)	−2.03 (0.05)^*^|^*^|^*^	−2.03 (0.05)^*^|^*^|^*^
international	−2.53 (0.02)^*^|^*^|^*^	−2.53 (0.02)^*^|^*^|^*^
international * Ins. type (Private Museum)	.10 (0.02)^*^|^*^|^*^	.10 (0.02)^*^|^*^|^*^
international * Ins. type (Public Institution)	−.22 (0.02)^*^|^*^|^*^	−.22 (0.02)^*^|^*^|^*^
international * Ins. type (Top-Tier Museum)	.23 (0.03)^*^|^*^|^*^	.23 (0.03)^*^|^*^|^*^
AIC	952025.9	952025.9
BIC	2519518.9	2519518.9
logLik	−355072.9	−355072.9
N	3143712	3143712
pseudo-R^2^	0.31	0.31

standard errors in parentheses.^***^p < 0.001;^**^p < 0.01;^*^p < 0.05.

**Fig 2:**
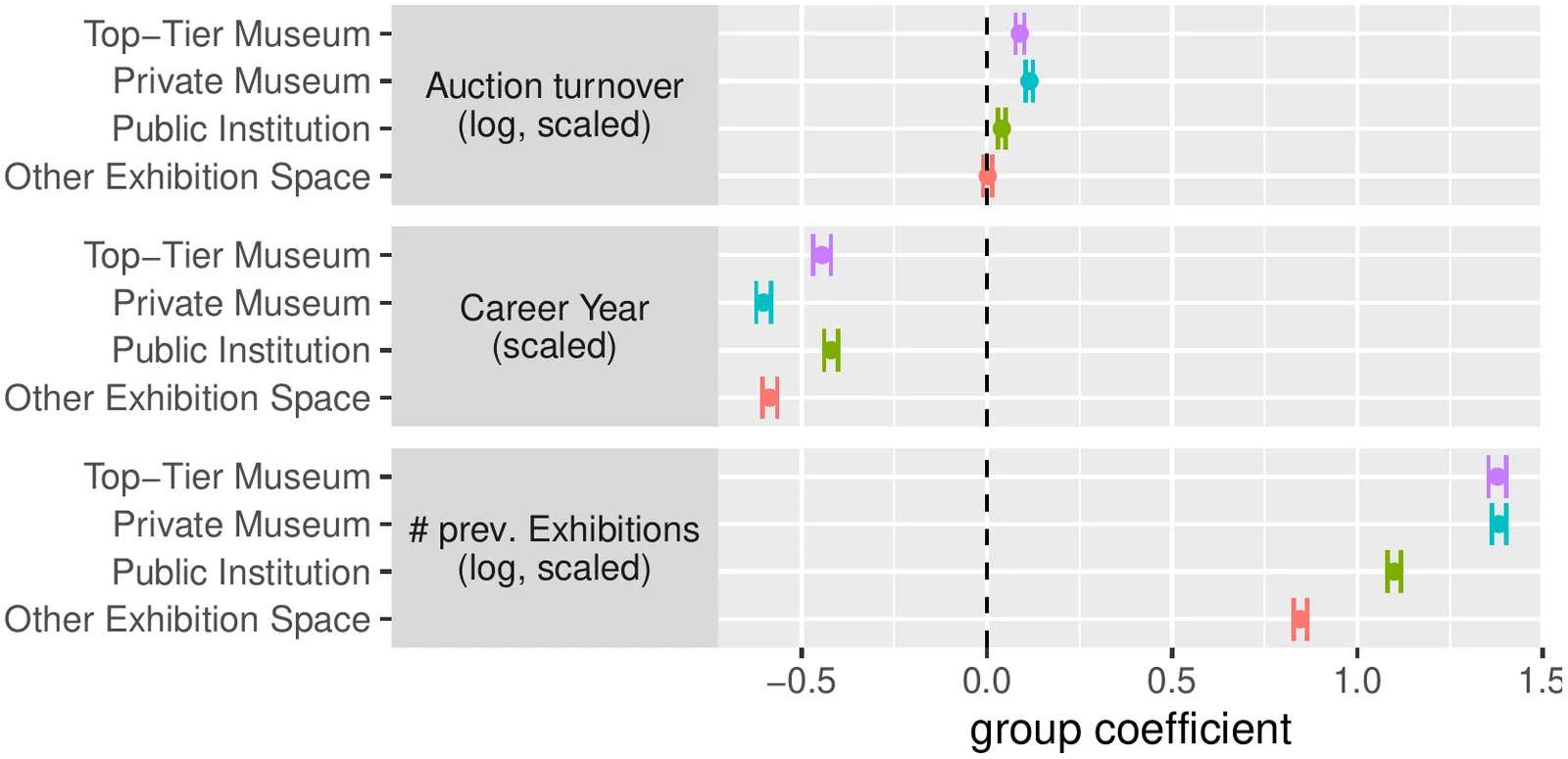
Slopes by institution type (scaled).

**Fig 3:**
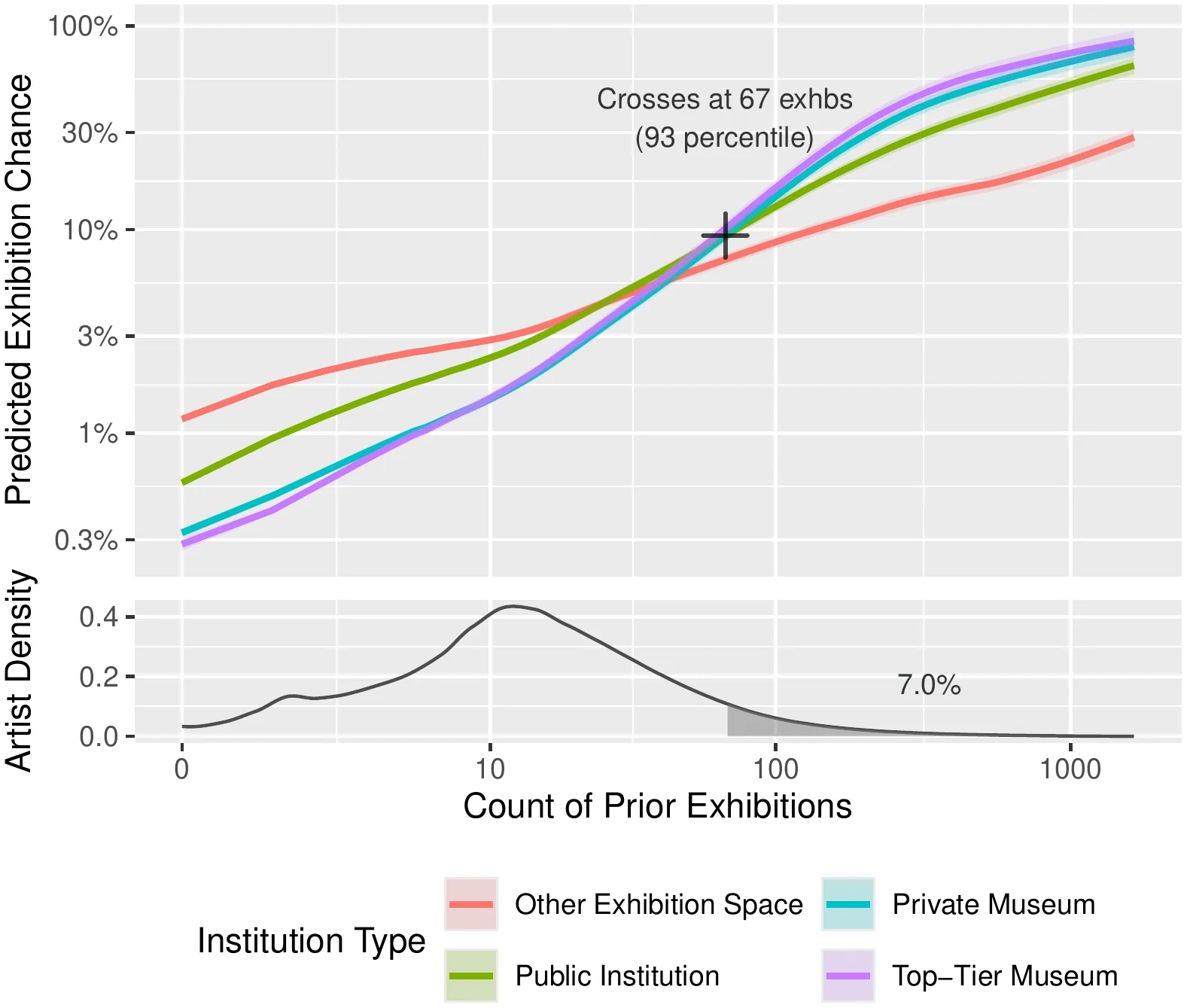
Average adjusted predictions by institution type and count of prior exhibitions.

The REM results show that prior exhibitions increase exhibition rates in all institution types (positive main effect and interactions). All institution types thus display a Matthew effect [61,62]. To situate this general tendency, it helps to see how exhibition chances vary over the range of observed prior exhibition counts for public institutions. In Table 4, we see that public institutions have a slope of around 1 (0.77 + 0.23) and thus provide a illustrative example: Let us assume that an artist counts as a superstar when they have been exhibited 1100 times (which has been achieved by only a handful of artists); the corresponding log of prior exhibition count is then around 7 (exp(7) ≈ 1100). Then, exhibition rates between the career starters and superstars similarly vary by a factor of exp(7) ≈ 1100, indicating that the superstars experience exhibition rates 1100 times higher than those of career starters (assuming all other variables are equal). Furthermore, the significant interactions show that the effect of prior exhibitions differs between institution types. It is substantially stronger in private museums (1.3) and top-tier museums (1.3) than in public institutions (1) and other exhibition spaces (0.77). Whereas exhibition rates between career starters and superstars in public institutions vary by a factor of ≈ 1100, in private museums (and top-tier museums) the variation is exp(1.3*7) ≈ 9000, thus indicating a substantially stronger preference for established artists in private and top-tier museums.

Beyond comparing variation in exhibition rates within institution types, we can compare exhibition chances more broadly by using Average Adjusted Predictions, i.e., calculating predictions for every observation in the dataset and averaging predicted exhibition rates by institution type and count of prior exhibitions (Fig 3). For ease of interpretability we use percentages rather than rates, the predicted quantity is thus the probability of being chosen out of the risk set of 26 artists (the absolute range of the vertical axis is thus heavily dependent on the number of controls, with larger numbers of controls resulting in lower predicted exhibition rates, i.e., a model based on higher numbers of control cases would show lines of same steepness at numerically lower predicted exhibition chances. Number of controls however does not affect the relative differences between institution types, the main focus of the figure). The positive slope of all lines indicates a preference for established artists effect in all institution types (an institution type without such preference would display a horizontal line at 100/26 = 3.84%, the random chance of each event). Variation in the steepness of the slope indicates variation in the strength of the preference for established artists, with private museums and top-tier museums having both a relatively steep slope and public institutions and other exhibition spaces displaying a shallower slope. Next to slope variation, it can be seen that at lower prior exhibition counts, artists have higher exhibition chances in other exhibition spaces and (to a lesser extent) public institutions than in private museums and top-tier museums, whereas this pattern first equalizes and eventually reverses at higher prior exhibition counts, with established and superstar artists having higher exhibition chances in top-tier and private museums than in public institutions and other exhibition spaces. The lower panel shows the distribution of prior exhibition count in the data set (event and risk set), and thus facilitates the interpretation of at which point artists experience changes in institutional exhibition rates. The crossing point of public institutions and private museums is marked with a cross, which indicates that artists with less than 67 exhibitions (which amounts to 93% of artists) have better prospects in the average public institution exhibition than the average private museum exhibition.

This pattern of variation in the preference for established artists is also present in the case of auction turnover. Auction turnover increases exhibition chances most strongly in private museums, followed by top-tier museums and public institutions. Auction turnover however (contrary to prior exhibition count) does not increase exhibition rates in other exhibition spaces (insignificant main effect, corresponding to a group slope including zero).

The REM coefficient of career year is negatively associated with exhibition chances in all institution types (Fig 2). Seemingly contrary to the descriptive findings, the overall negative effect is compatible with a preference for successful artists: REM estimates the effect of gaining a career year without achieving additional exhibitions or auction turnover (as these other variables are held constant), therefore the negative career-year effects represent penalties for failing to attract recognition: If we imagine artist A and B who have the same number of prior exhibitions and auction turnover, but artist A has achieved these accomplishments in 5 years whereas artist B needed 10, artist B would experience lower exhibition rates, presumably for having achieved recognition at a lower pace than artist A. Variation of this effect is however differently distributed than those in prior exhibitions and auction turnover. Whereas both other variables have the strongest effects in private museums and top-tier museums, and weaker effects for public institutions and other exhibition spaces, here the strongest effects are observed in private museums and other exhibition spaces, whereas effects are weaker in top-tier museums and public institutions. While it is not clear why private museums and top-tier museums diverge on this variable, it provides further evidence of private museums strongly prioritizing artists who are receiving recognition at high speeds.

In Model 2, the variables are scaled to a standard deviation of 1 to compare effect sizes (Fig 2 also uses the standardized variables for the institution type slopes). This reveals that the count of prior exhibitions has the largest effect, followed by career year. Auction performance, while still significant, influences exhibition chances comparatively little. These patterns are also observed when investigating the contribution of each variable to model improvement (see supplementary materials). The fact that exhibition choices are primarily driven by prior exhibition choices rather than auction performance, especially in other exhibition spaces, indicates a certain independence of the artistic valuation from the art market.

In terms of control variables, a strong negative overall effect of international artists reflects the national organization of art fields. This effect is strongest in public institutions, which might be required to support national art scenes when receiving government funding [43], and weaker in private museums (possibly due to independence from national funding bodies and global orientation of their founders) and top-tier museums (who might be incentivized to show international well-known artists to reach large audience numbers). Private museums, in line with the descriptive findings, still show a higher tendency to exhibit male artists than all other institution types (positive and significant interaction between Gender – male and institution type Private museum). Having exhibited at an institution previously strongly increases exhibition chances in all institution types, particularly in other exhibition spaces and private museums, indicating that other exhibition spaces and private museums repeatedly exhibit the same artists.

## Discussion and conclusion

In academic studies on private museums, two mutually exclusive perspectives exist on how private art museums program their exhibitions. On the one hand, scholars have argued that private museums tend to show underexposed, emerging art due to the influence of a visionary and independent founder. On the other hand, scholars have claimed that private museums merely reinforce the reputation of established artists since private collectors are heavily oriented towards existing hierarchies of symbolic and commercial valuation. In this paper we have provided the first systematic empirical analysis of the exhibition choices of private museums relative to other institutional forms. Our results show a tendency for private museums to exhibit established artists at greater rates than public institutions and other exhibition spaces. These private museums have a strong taste for consecrated artists, most similar to that of global top-tier museums, thus revealing that this new organizational form usually does not embrace novelty-oriented programming. We separately analyzed the similarity between institutional forms by calculating exhibition choice overlap, i.e., the extent to which institutional forms exhibit the same artists (see supplementary materials). Our results show that the observed overlap between the portfolios of private museums and top-tier museums is around 4.4 times stronger than would be expected by random chance alone. This overlap is much stronger than between any other pair of institutions, indicating that private museum exhibition patterns correspond most closely to those of established elite institutions.

The fact that private museum exhibition patterns are most similar to those of a small number of top-tier museums is particularly noteworthy, as proponents of private museums as novelty-oriented organizations have identified resistance against (or at least divergence from) these top-tier museums (and their programming “where everything has to be popular” [37]) as private museums’ raison d’être. The particular mechanism behind such convergence is beyond the scope of this paper. We consider the most likely explanation to be the influence of their founders, who appear to be (as suggested by scholars critical of private museums’ innovative potential) less interested in novel perspectives and rather prefer to exhibit already consecrated artists with high institutional and commercial recognition. Following the exhibition choices of highly prestigious organizations could also be a case of mimetic isomorphism [74], i.e., through exhibiting well-known artists private museums would be able to acquire the status of legitimate elite institutions themselves.

At the same time, alternative explanations cannot fully be ruled out: Collector influence in both private and top-tier museums might result in programming convergence, as collectors often sit on the boards of directors of various institutions; however, top-tier museums programming involves wide variety of other actors [75], making it unlikely that collectors would be able to impose their preferences as thoroughly as in their private museums. Concerns about visitor numbers might also play a role, but are unlikely the primary driver as entrance fees constitute a relatively small proportion of earned income [45,76]. While therefore other factors are certainly not negligible, we consider it most likely that the exhibition choices of private museums (especially given these institutions’ financial and regulatory independence) primarily reflect the preferences of the individuals who govern and finance them, i.e., their founders.

The other striking finding of this study is that private museums are more likely than other institutions to exhibit artists with an active auction turnover. This seems to confirm previous studies which have hypothesized that the curatorial choices are informed by market considerations: they may follow the advice of dealers or auctioneers, or may select ‘blue chip’ artists as a form of conspicuous consumption or for financial investment reasons [15]. At the same time, also in this respect top-tier museums resemble private museums to some extent, contradicting previous studies which have claimed that such museums steer away from commercially successful artists [77]. The exact sequence of events may be crucial here, however: it may be the case that the artists become blue chip on the market because of the consecration by top-tier museums, and only after this consecration and market stabilization has taken place, private museums take an interest in these artists. In order to understand this process in more depth, future research on the impact of curatorial decisions on artists’ careers and market success is needed.

There are many other opportunities for future research to develop these insights further. First, investigating the mechanisms of the curatorial process qualitatively would advance our understanding of how institutions vary in their evaluations and programming of artists. Secondly, rather than focusing exclusively on inter-form variation, variation of exhibition patterns can be investigated in many other aspects, such as between other organizational dimensions or national contexts, or between exhibition formats, such as exhibition length. Thirdly, further insights into curatorial decision making could also be gained from additional data sources, such as the permanent collection of institutions (of which usually only a small part is on show, see, e.g., [78] for variation in collection acquisition patterns), artwork-level exhibition data (thus enabling the incorporation of aesthetic criteria such as styles, or more elaborate artist characteristics such as awards or ethnicity.

Finally, this study provides a more nuanced understanding of the wider role of private museums as an exemplary case of elite cultural philanthropy. Their tendency to show established artists is providing clear benefits to audiences who like to see such art and are unlikely to visit exhibitions of less established artists. Following the exhibition choices of the most famous museums in particular might solidify their image. Furthermore, by making otherwise inaccessible artworks accessible, private collectors may enhance access especially in (semi-)peripheral regions of the Global South where cultural service provision by the state or other actors is limited. At the same time, however, a sustainable art ecosystem requires innovation, such as support for emerging artists. If private museums do not fulfill this role, despite being well situated for it given their substantial resources and independence from political agendas, and engage primarily in what some see as conspicuous consumption, it is not unimaginable that the public benefit of these organizations will be called into question.

## Supporting information

S1 Appendix Supporting Information. (PDF)
