# Supplementary material for: Bandwagons or breeding grounds for innovation: What economic elites’ private museums contribute to the arts
Source: PLoS One. 2026 Oct 9;21(10):e0360158. doi: 10.1371/journal.pone.0360158 (PMC13652257; doi:10.1371/journal.pone.0360158)
Supplement: Supplementary file 1 [file pone.0360158.s001.pdf]

S1 Appendix

REM technical details

On a technical level, REM compares observed events to events that could have taken place (Lerner & Lomi, 2020): Given a sequence of events $E = (e_1, \dots, e_N)$, each event can be formulated as $e_i = (u_i, v_i, t_i)$, i.e. taking place at time t_i between sender u_i and receiver v_i. The hazard rate (or intensity or event rate) of a dyad can then be defined as:

$$\lambda(u, v, t) = \lim_{\Delta t \to 0} \frac{P(t \leq T \leq t + \Delta | t \leq T)}{\Delta t}$$

where T denotes the time of the next event on (u, v). The hazard is specified as

$$\lambda(u, v, t, G[E; t]; \theta) = \lambda_0 \cdot \lambda_1(u, v, t, G[E; t]; \theta),$$

where $\lambda_0(t)$ is a time-varying baseline hazard, modified by the relative event rate λ_1, which "is proportional to the probability that an event at t happens on (u, v), rather than on any other dyad in the risk set" (Lerner & Lomi, 2020, p.100), and defined as

$$\lambda_1(u, v, t, G[E; t]; \theta) = \exp\left(\sum_{l=1}^{k} \theta_l \cdot s_l(u, v, G[E; t])\right)$$

where θ constitutes the parameter vector of $s(u, v, G[E; t])$, the sufficient statistics based on node, dyad and network characteristics (either time-invariant or based on past events). θ constitutes the main quantity of interest, as it describes which characteristics increase or decrease the probability of events occurring, and can be estimated via partial likelihood maximization:

$$L(\theta) = \prod_{e_i \in E} \frac{\lambda_1(u_i, v_i, t_i, G[E; t_i]; \theta)}{\sum_{uv \in R_{t_i}} \lambda_1(u, v, t_i, G[E; t_i]; \theta)}$$

with R_{t_i} as risk set, i.e. the set of dyads on which event e_i could have been observed but was not.

Model improvement by variable

Figure 1 shows how the addition of each main variable affects the fit of the relational event model. Three scenarios are investigated. First, each variable is added to an empty model (column 1). Secondly, each variable is added to a model with the control variables (gender and international exhibition, column 2). Thirdly, each variable is added to a model that contains each variable but the focal one (column 3). It can be seen that count of previous exhibitions in all scenarios results in the greatest model improvement; while the improvements of adding the other variables while mostly still substantial are smaller and more dependent on the scenario.

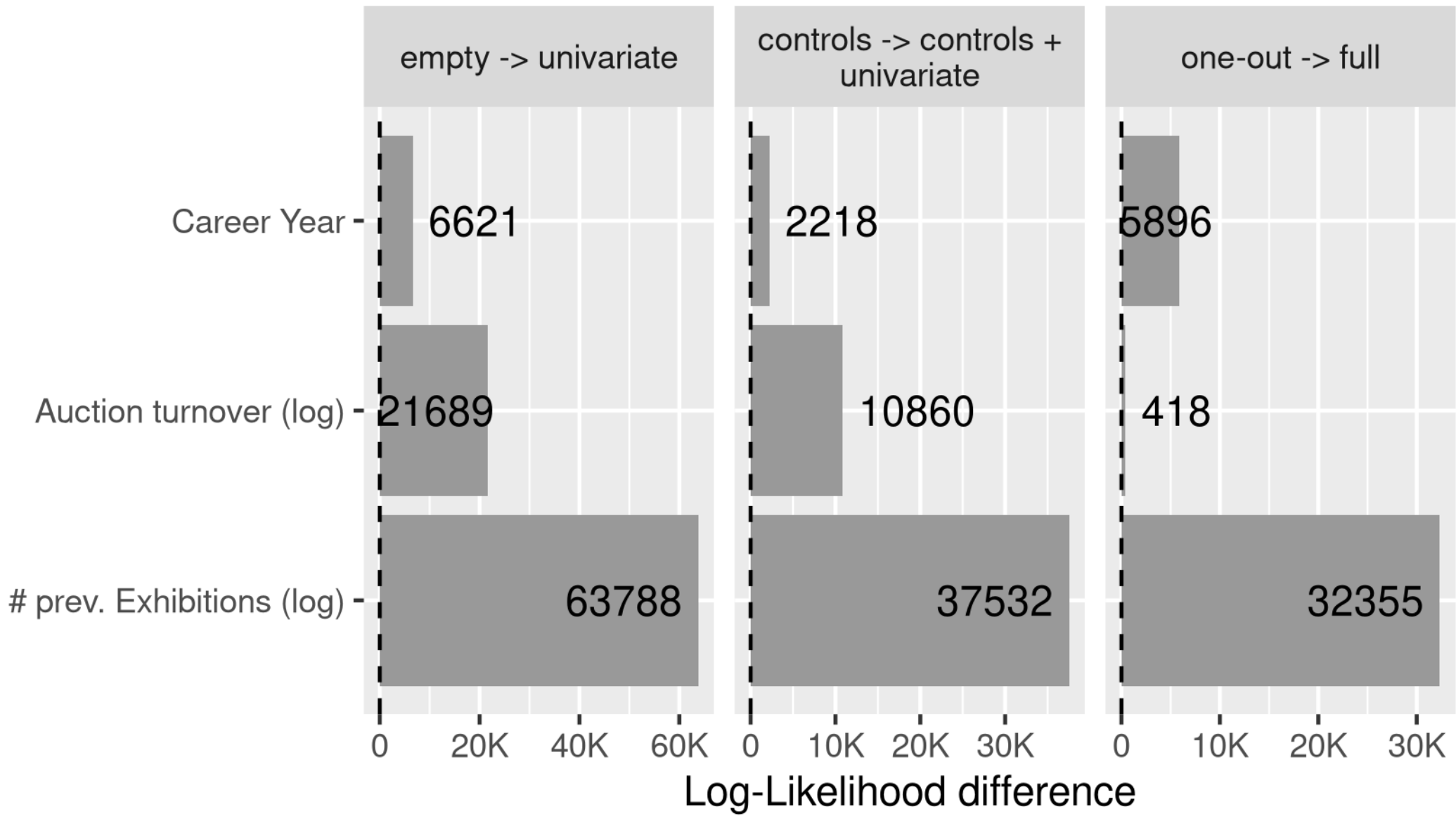

Figure 1: Model improvements by variable

Additional models

Variable	m3	m4	m5	m6	m7
**all**					
# prev. Exhibitions (log)		.51 (0.01)***			.77 (0.01)***
# prev. Exhibitions (log) * Ins. type (Private Museum)		.57 (0.01)***			.49 (0.01)***
# prev. Exhibitions (log) * Ins. type (Public Institution)		.33 (0.01)***			.23 (0.01)***
# prev. Exhibitions (log) * Ins. type (Top-Tier Museum)		.63 (0.01)***			.49 (0.01)***
Auction turnover (log)			.05 (0.00)***		.00 (0.00)
Auction turnover (log) * Ins. type (Private Museum)			.10 (0.00)***		.04 (0.00)***
Auction turnover (log) * Ins. type (Public Institution)			.06 (0.00)***		.01 (0.00)***
Auction turnover (log) * Ins. type (Top-Tier Museum)			.10 (0.00)***		.03 (0.00)***
Career Year				−.01 (0.00)***	−.07 (0.00)***
Career Year * Ins. type (Private Museum)				.04 (0.00)***	−.00 (0.00)
Career Year * Ins. type (Public Institution)				.03 (0.00)***	.02 (0.00)***
Career Year * Ins. type (Top-Tier Museum)				.05 (0.00)***	.02 (0.00)***
Gender - Male	−.13 (0.01)***	−.20 (0.01)***	−.15 (0.01)***	−.11 (0.01)***	−.13 (0.01)***
Gender - Male * Ins. type (Private Museum)	.26 (0.02)***	.18 (0.02)***	.17 (0.02)***	.18 (0.02)***	.17 (0.02)***
Gender - Male * Ins. type (Public Institution)	.10 (0.02)***	.05 (0.02)*	.06 (0.02)**	.02 (0.02)	.03 (0.02)
Gender - Male * Ins. type (Top-Tier Museum)	.11 (0.02)***	.05 (0.02)*	.02 (0.02)	.01 (0.02)	.02 (0.02)
Gender - Other	.43 (0.06)***	.18 (0.06)**	.44 (0.06)***	.43 (0.06)***	.10 (0.06)
Gender - Other * Ins. type (Private Museum)	.19 (0.08)*	−.04 (0.09)	.30 (0.08)***	.19 (0.08)*	.02 (0.09)
Gender - Other * Ins. type (Public Institution)	.02 (0.08)	−.10 (0.08)	.07 (0.08)	.01 (0.08)	−.06 (0.08)
Gender - Other * Ins. type (Top-Tier Museum)	.16 (0.09)	.02 (0.09)	.33 (0.09)***	.17 (0.09)	.08 (0.09)
Repeated exhibiton	4.26 (0.03)***	4.00 (0.03)***	4.23 (0.03)***	4.28 (0.03)***	4.02 (0.04)***
Repeated exhibiton * Ins. type (Private Museum)	.39 (0.05)***	−.38 (0.05)***	.07 (0.05)	.25 (0.05)***	−.42 (0.05)***
Repeated exhibiton * Ins. type (Public Institution)	−.63 (0.04)***	−1.16 (0.05)***	−.82 (0.05)***	−.76 (0.05)***	−1.15 (0.05)***
Repeated exhibiton * Ins. type (Top-Tier Museum)	−.76 (0.04)***	−2.07 (0.05)***	−1.33 (0.05)***	−1.05 (0.05)***	−2.03 (0.05)***
international	−2.47 (0.01)***	−2.49 (0.01)***	−2.48 (0.01)***	−2.48 (0.01)***	−2.53 (0.02)***
international * Ins. type (Private Museum)	.22 (0.02)***	.10 (0.02)***	.18 (0.02)***	.22 (0.02)***	.10 (0.02)***
international * Ins. type (Public Institution)	−.11 (0.02)***	−.23 (0.02)***	−.14 (0.02)***	−.11 (0.02)***	−.22 (0.02)***
international * Ins. type (Top-Tier Museum)	.43 (0.02)***	.23 (0.03)***	.43 (0.02)***	.45 (0.02)***	.23 (0.03)***
AIC	1039179.2	964124.1	1017467.1	1034751.1	952025.9
BIC	2606516.7	2531513.5	2584856.4	2602140.5	2519518.9
logLik	−398661.6	−361130.1	−387801.5	−396443.6	−355072.9
N	3143712	3143712	3143712	3143712	3143712
pseudo-R^2	0.23	0.30	0.25	0.23	0.31

standard errors in parentheses. ***$p < 0.001$; **$p < 0.01$; *$p < 0.05$.

Additional models that vary in the inclusion of the main independent artist career variables are reported in table 1, showing a number of noteworthy findings. Firstly, career year (in all institution types but other exhibition spaces) still has a positive effect when previous exhibitions and auction turnover are not controlled for. Secondly, the coefficients of auction performance shrinks strongly by the addition of other variables measuring the degree of artist establishment, whereas the effect of count of previous exhibition increases.

Exhibition choice overlap

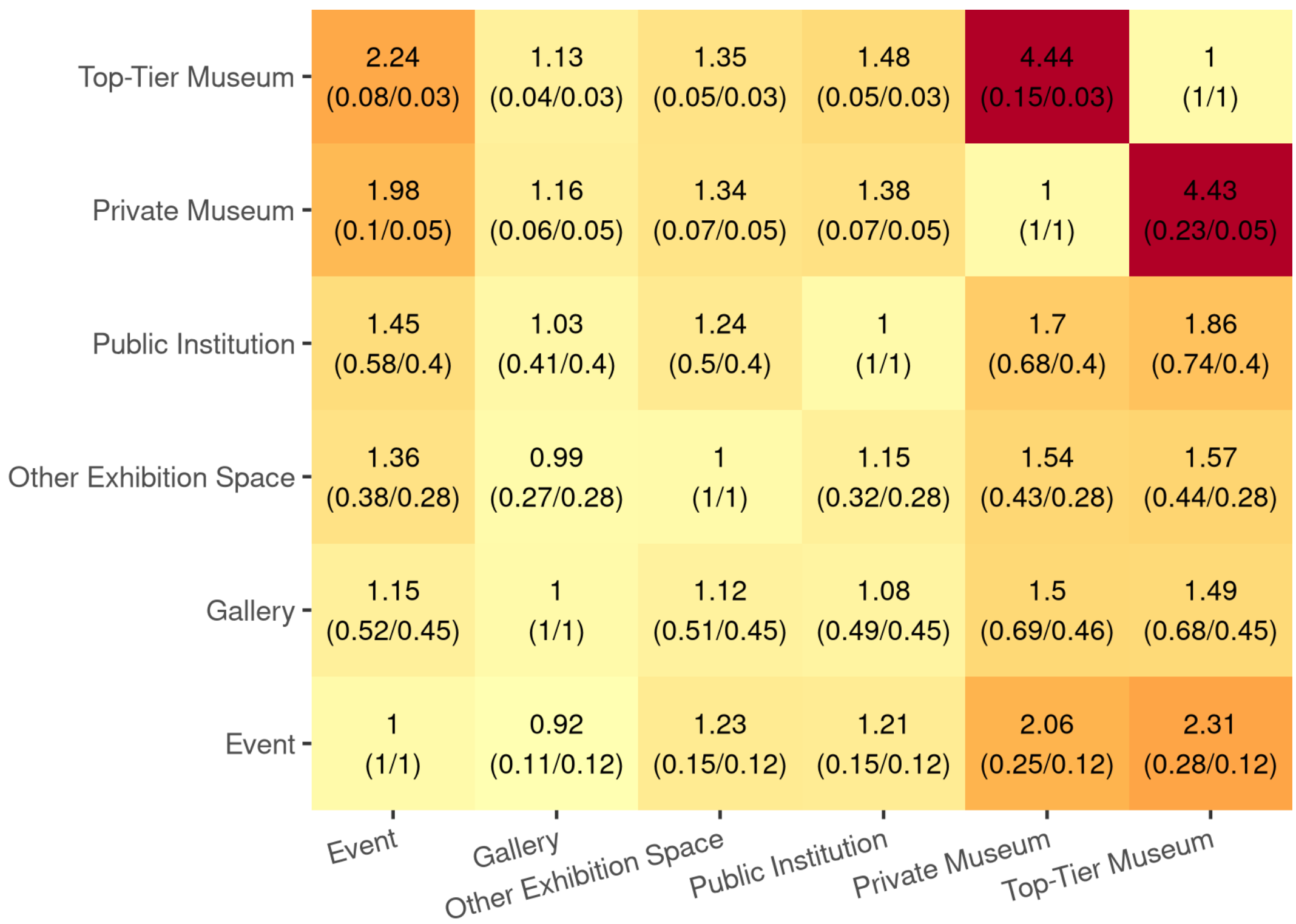

Figure 2: Overlap between institution types

Next to investigating how similar the portfolios of different institutional forms are in terms of the career characteristics of their respective artists, we also analyze the similarity of exhibition choices by analyzing choice overlap, i.e. comparing how similar portfolios are compared to how similar they would be if choices were independent. For this, we first calculate the observed (asymmetric) overlap coefficient between the choice sets of forms A and B as $|A \cap B| / |A|$ for all pairwise institution combinations per year, and then average these pairwise overlap proportions across years. For example, on average 15% of artists shown in private museums also have an exhibition in a top-tier museum in the same year, and 23% of artists in top-tier museums are shown in private museums. However, these absolute numbers are heavily driven by group size (e.g. 74% of artists in top-tier museums also have public institution exhibitions, yet comparing the top-tier-public institution overlap of 74% to the top-tier-private museum overlap of 23% is not insightful because there are many more public institutions than private museums). We

therefore divide this observed overlap coefficient by the expected overlap if exhibition choices were made independently (obtained by simulating the same number of exhibition choices as observed but with each choice assigned to a random artist). Returning to the overlap of private museums and top-tier museums, we find that (since top-tier museums are a small group) only 3% of private museum artists would (on average) be expected to be shown in top-tier museums in the same year if choices were made independently. This means that the observed 15% overlap is around 4.4 times stronger than would be expected by random chance alone. Figure 2 shows all pairwise overlaps. While most relative overlaps are larger than 1 (due to general concentration of exhibitions in a small group of artists), the relative overlap measures between private museum and top-tier museums are by far the strongest, indicating again that private museum exhibition patterns correspond most closely to those of established elite institutions.

Alternative top-tier specifications

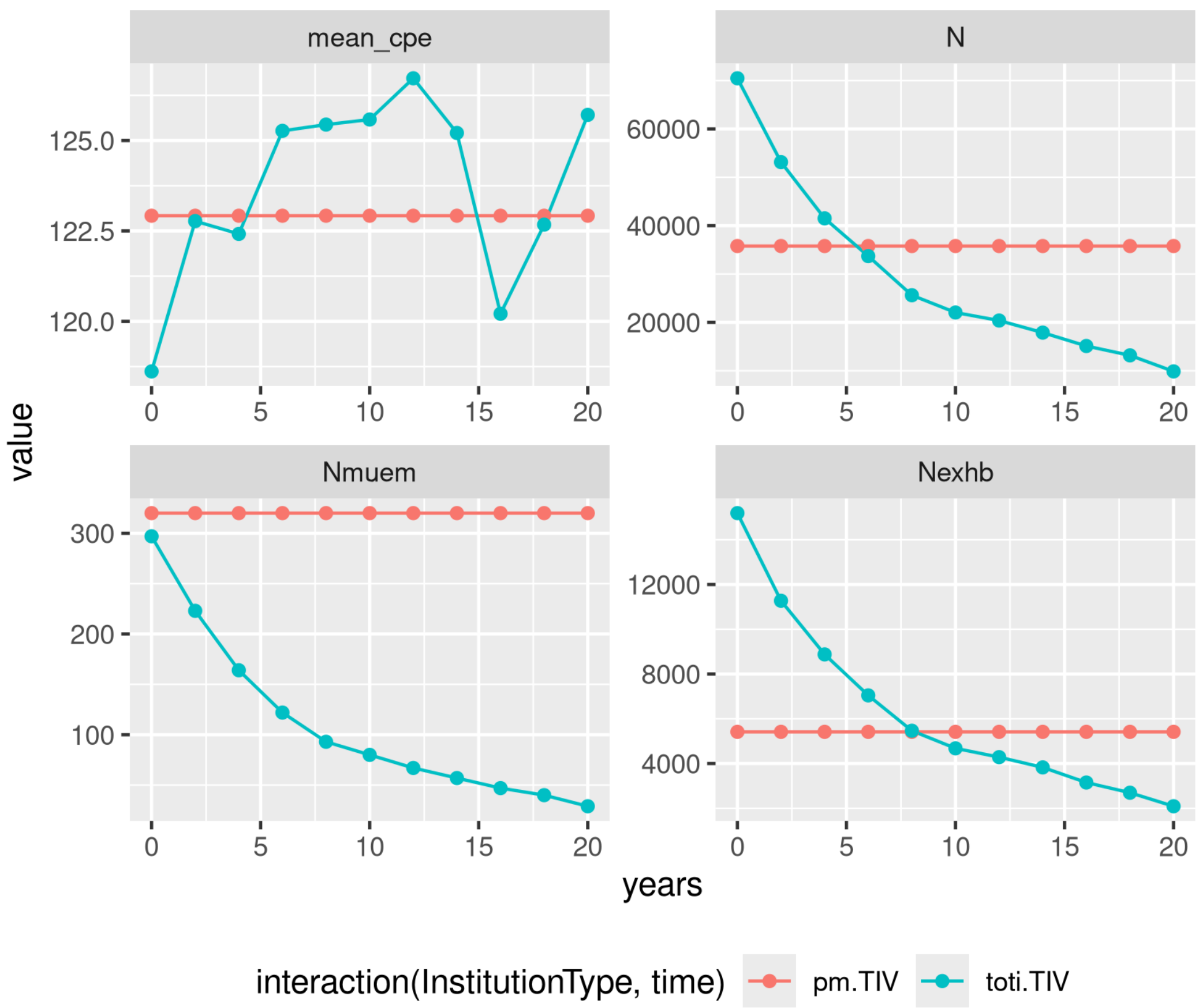

Figure 3: variation in mean CPE by instype and toti specification

In the main analysis, institutions are defined as top-tier if they appear at least 10 times in the Art Newspaper's annual museum ranking. We also test alternative operationalizations, in particular we vary the number of years that are required to be classified as a top-tier institution from 1 (all organizations that are in the Art Newspaper ranking are classified as top-tier) to 20

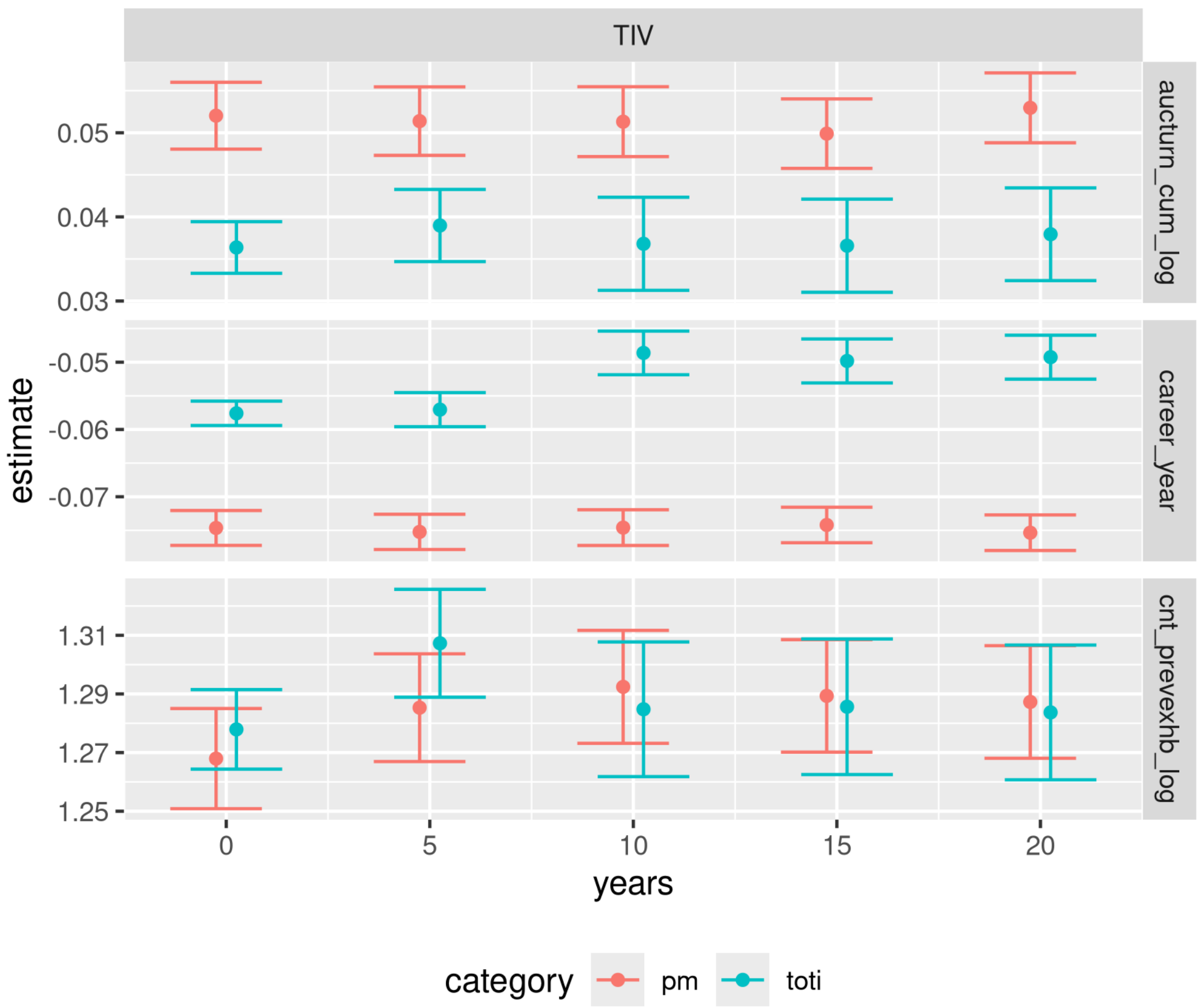

Figure 4: group slopes (PM vs toti) depending on toti specification

(only institutions that almost always appear count as top-tier) in 5 year steps. Number of years (expectedly) substantially affects number of institutions, exhibitions and artists-exhibition links (figure 3). However, coefficients on the main variables show overall relatively little variation across specifications (figure 4). We interpret this as the presence of an artistic canon at the top museums which is exhibited relatively evenly among top-tier institutions.

Inverse Cluster Size Weights

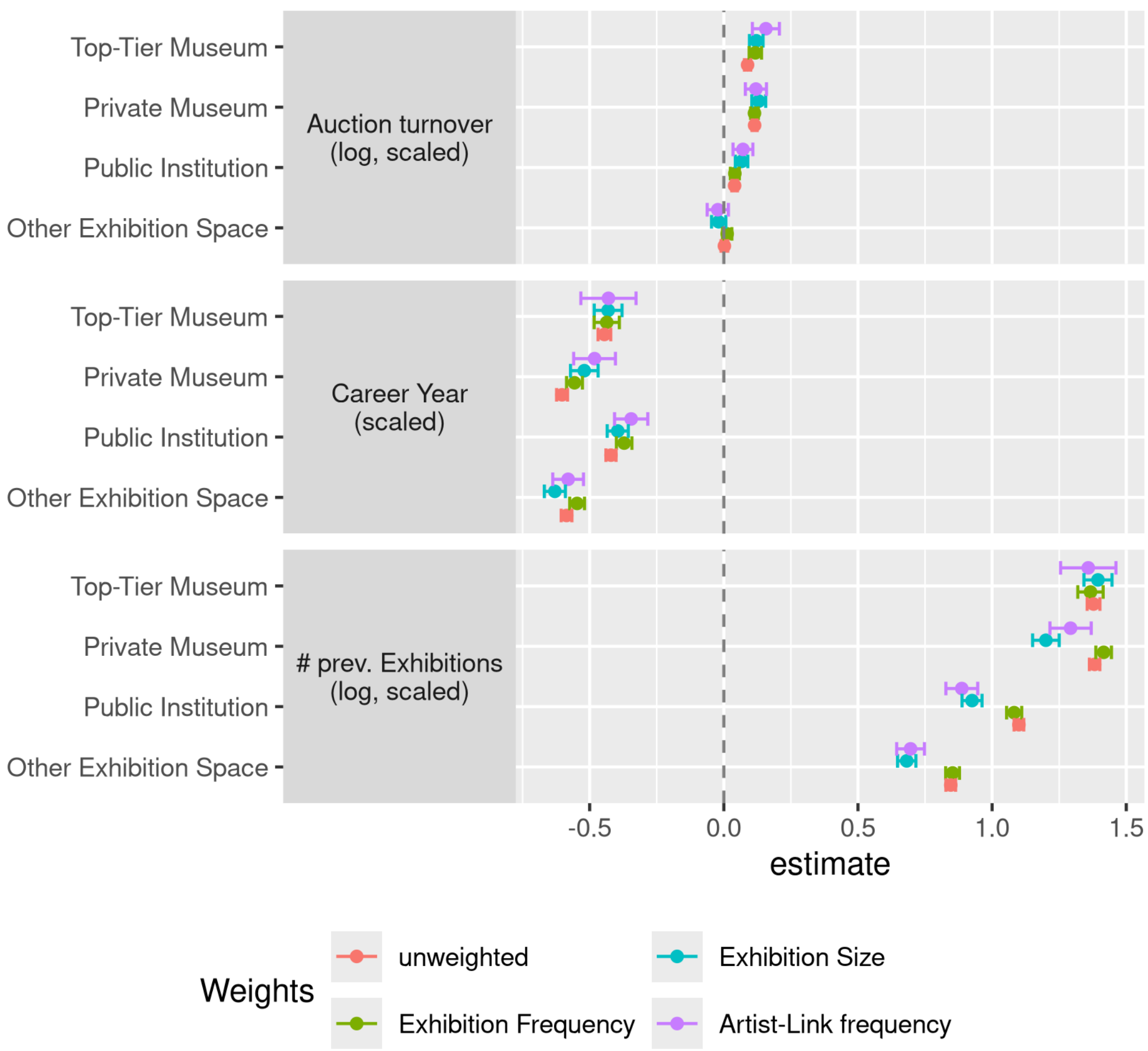

Figure 5: Institution slopes under different weights

We explore how coefficients change under different weights, to investigate how stable results are to institution-(type) specific patterns regarding exhibition size and frequency. We use inverse-cluster size weights, which decrease the weight of observations (exhibition choices) inversely by the size of the cluster, so that each cluster contributes the same amount of information to the model. We investigate three kinds of clusters:

- Exhibition size: Exhibition choices are weighted by the number of artists included in an exhibition, i.e. exhibition choices in a large exhibition contribute less than those in a small, or solo exhibitions.

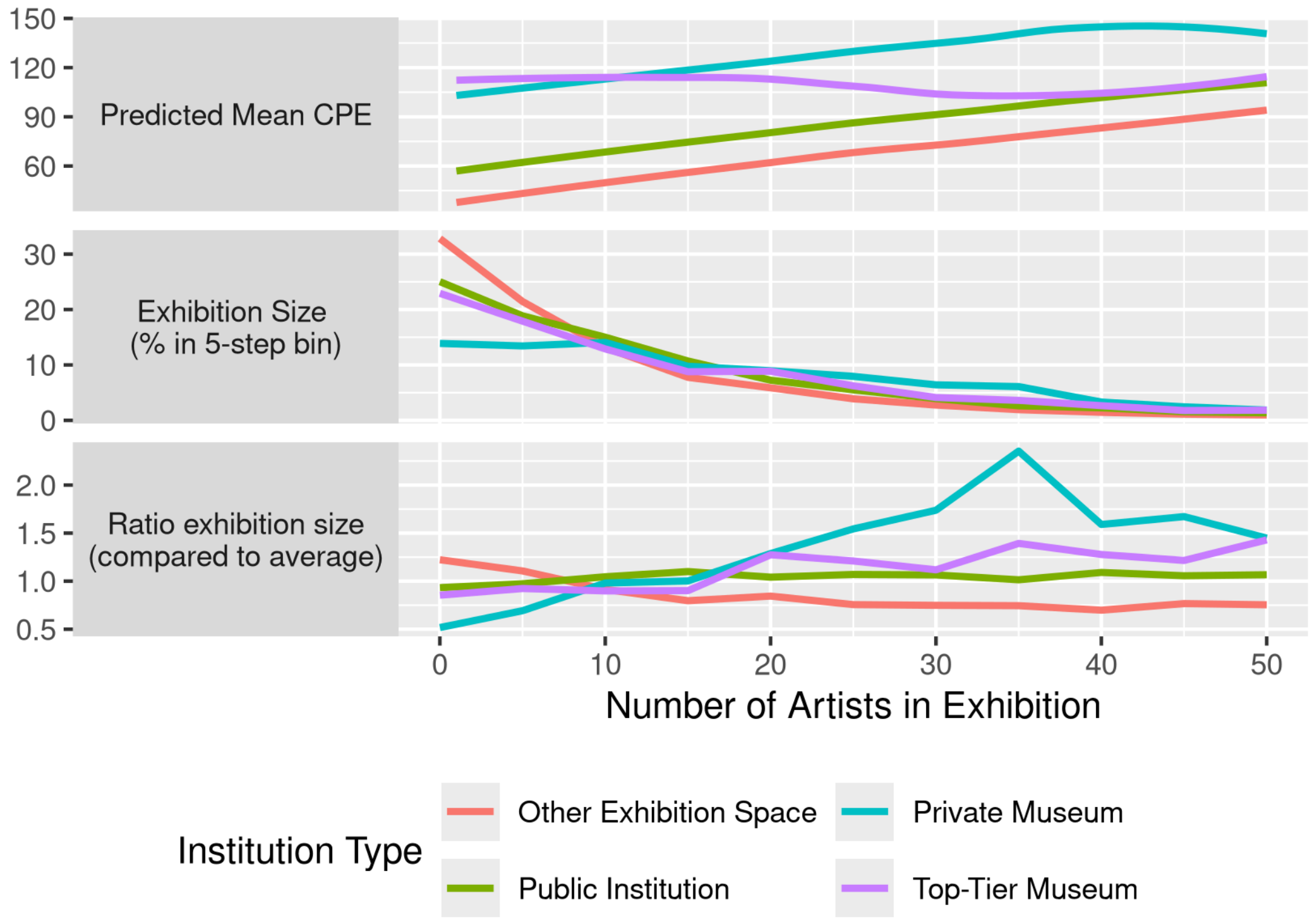

Figure 6: Descriptives of exhibition size

- Exhibition Frequency: Exhibition choices are weighted by the number of exhibitions an institution organizes in the year in question, i.e. exhibition choices of institutions that organize many exhibitions in a year are given less influence.
- Artist-Link Frequency: Exhibition choices are weighted by the total number of exhibition choices an institution makes in a year, which can come from either organizing large exhibitions or organizing many exhibitions. Under this cluster, each institution-year contributes the same amount of information.

Slopes of the models are shown in figure 5, showing overall no substantial divergences of the weighted models from the unweighted models. Minor divergences are present for exhibition choices weighted by exhibition size, in which case private museums and top-tier museums differ significantly in the effect size of prior exhibition count (however, the total difference is relatively small, and significantly primarily due to the large sample size). Further investigation shows that this tendency is due to private museums organizing disproportionately large shows, and in these large shows show disproportionately established artists (figure 6). It is likely the that decreased weight of these large shows (in the exhibition-size weight) leads to a slightly decreases coefficient; however the overall patterns of private museums showing relatively established artists remains generally stable.

References

References

Lerner, J., & Lomi, A. (2020). Reliability of relational event model estimates under sampling: How to fit a relational event model to 360 million dyadic events. *Network Science*, *8*(1), 97–135. https://doi.org/10.1017/nws.2019.57
